# Feasibility and accuracy of continuous intraabdominal pressure monitoring with a capsular device in human pilot trial

**DOI:** 10.1186/s13017-024-00569-0

**Published:** 2025-01-27

**Authors:** Chien-Hung Liao, David A. Spain, Chih-Chi Chen, Chi-Tung Cheng, Wei-Cheng Lin, Dong-Ru Ho, Heng-Fu Lin, Fausto Catena

**Affiliations:** 1 Department of Trauma and Emergency Surgery, Chang Gung Memorial Hospital, Chang Gung University, Taoyuan, Taiwan; 2 Department of Surgery, Stanford University, Stanford, CA USA; 3 Department of Physical Medicine and Rehabilitation, Chang Gung Memorial Hospital, Chang Gung University, Taoyuan, Taiwan; 4 Department of Electrical Engineering, Chang Gung University, Taoyuan, Taiwan; 5 Department of Surgery, Division of Urology, Chang Gung Memorial Hospital, Chang Gung University, Puzi City, Chiayi, Taiwan; 6 Division of Trauma, Department of Surgery, Far Eastern Memorial Hospital, New Taipei City, Taiwan; 7 University of Bologna- Bufalini Hospital, Cesena, Italy; 8 Graduate Institute of Medicine, Yuan Ze University, Taoyuan City, Taiwan; 9 School of Medicine, National Tsing Hua University, Hsinchu, Taiwan

**Keywords:** Intraabdominal pressure, Abdominal compartment syndrome, Capsular sensor, Digital health

## Abstract

**Background:**

Intrabdominal pressure (IAP) is an important parameter. Elevated IAP can reduce visceral perfusion, lead to intraabdominal hypertension, and result in life-threatening abdominal compartment syndrome. While ingestible capsular devices have been used for various abdominal diagnoses, their application in continuous IAP monitoring remains unproven.

**Method:**

We conducted a prospective clinical trial to evaluate the feasibility of IAP measurement using a digital capsule PressureDOT, an ingestible capsule equipped with wireless transmission capability and a pressure sensor, then compared its reliability with conventional intravesical method. Patients undergoing laparoscopic or robotic surgeries were recruited. During surgery, we created pneumoperitoneum by inflating CO2 into the peritoneal cavity and IAP was simultaneously monitored using both the ingestible capsules and intravesical measurements from Foley catheter. We assessed the feasibility of signal transmission and the accuracy of pressure measurements.

**Results:**

Six patients were enrolled in this pilot study. No adverse events were reported, and the average first-intake time was within 24 h. All capsules were successfully expelled, with an average excretion time of 81 h. In the summarized data, the mean IAPdot is 0.6 mmHg lower than the IAPivp, with a standard deviation of 1.68 mmHg. However, capsule measurements showed excellent correlation with intravesical IAP measurements, with an intraclass correlation coefficient of 0.916 (95% CI: 0.8821–0.9320).

**Conclusion:**

Our study demonstrates the feasibility and safety of using digital capsules for continuous IAP monitoring, providing the agreement between IAP measurements from digital capsules and conventional intravesical measurement within a near-normal pressure.

**Supplementary Information:**

The online version contains supplementary material available at 10.1186/s13017-024-00569-0.

## Introduction

Intraabdominal pressure (IAP) is the constant pressure present in the closed abdominal cavity [1, 2]. IAP varies in the range of 5–7 mmHg in the healthy population [3, 4]. In people whose abdominal girth gradually increases, such as in obesity, pregnancy, or ascites, the IAP values can elevate to 10–15 mmHg without leading to harmful effects [5–7]. Abdominal perfusion pressure (APP) [8], which was defined as the difference between the mean arterial pressure and IAP [9], has been proposed as a more accurate predictor of visceral perfusion and consequently a target for intervention. Intraabdominal pressure is an essential parameter monitored in the critical care units. The visceral perfusion is highly associated with the change of IAP over time. For patients with an acute increase in IAP causes a drop in APP that leads to haemodynamic changes that can result in significant organ dysfunction and may lead to increasing morbidity and mortality [10]. Intraabdominal hypertension (IAH) is defined as a continuous rise in IAP above 12 mmHg. The WSACS consensus definition varies slightly and is as follows: Grade I (12–15 mmHg); Grade II (16–20 mmHg); Grade III (21–25 mmHg); Grade IV (> 25 mmHg) [1]. The abdominal compartment syndrome (ACS) is defined as a continuous IAP above 20 mmHg with newly developed organ dysfunction [11]. Untreated ACS is an independent predictor of organ failure and mortality [12, 13] that is often difficult to reverse, and should be prevented using different strategies. High mortality rate was noted when IAH and ACS took place [14–18]. and delayed decompression may not reverse the sequelae of IAH and ACS [19]. Therefore, close monitoring of IAP and early detection for IAH and prevention of further ACS is an important issue in critical care [20].

Currently, intravesical pressure (IVP) measurement is the gold standard for monitoring IAP. It is widely accepted as routine practice to measure intermittent IAP via the bladder every 4 to 6 h in symptomatic patients or those with a high clinical suspicion of developing IAH and ACS [10]. However, low accuracy, and high staff- dependent reliability and heavy workload were drawbacks for routine performing IVP measures in clinical practice [21–23].

Ingestible capsular sensors have long been recognized as valuable tools for real-time monitoring of intra-abdominal physical parameters [24–27]. Over the past decade, there have been substantial advancements in the design and functionality of ingestible electronic pills, particularly at the sensor, circuit, and system levels. These advancements have significantly enhanced the clinical utility of the technology by improving device sensitivity, extending operational lifespan, and increasing spatial accuracy [28]. Recent advances in the field of ingestible sensing [24, 25] have led to the development of ingestible capsule capable of continuously and accurately measuring pressure throughout the entire GI tract with minimal discomfort to the patient [29]. Although the concept of monitoring internal physiological conditions and pressure from within the body is well-established, the application of this technology specifically for IAP monitoring remains relatively rare in practice.

Digital pressure capsules for IAP monitoring and IAH detection have been developed [30]. The device consists of a piezoelectric sensor module with a low power wireless transmitter encased in a biocompatible capsule. Once activated and ingested, it allows for non-invasive IAP monitoring via wireless signal transmission, eliminating the need for invasive procedures. Previous studies showed the feasibility [31] and comparison results [30] in animal models. However, there was no study in human presentation. In this pilot study, we would like to perform the first in human study to validate its feasibility and efficiency to monitor IAP for patients.

## Materials and methods

### Recruited patients’ criteria

This study was conducted in Chang Gung memorial hospital (CGMH). We recruited patients who scheduled for laparoscopic and robotic surgery that may involve alterations in intra-abdominal pressure by CO2 inflation of peritoneal cavity during operation. The principal investigators and research team members explain the study protocol to the participants. All of the participants well informed and signed consents were included into this study. This study was approved by the institute research board of CGMH IRB No: 202301590A0.

Inclusion criteria included adults aged between 20 and 55 years with a BMI between 15 kg/m² and 35 kg/m². And patients’ general condition was adequate for safe anesthesia and operation. Patients will be excluded from the study if they have a high risk for capsule retention, such as intestinal diverticula, acute abdominal pain without regular defecation indicating intestinal obstruction, or a history of abdominal or intestinal reconstructive surgery. Exclusion also applies to patients with evidence of gastrointestinal tract occlusion or severe paralytic ileus requiring immediate surgical intervention. Patients who need to undergo an MRI examination within 7 days after ingestion of the capsule are also excluded. Female patients who are pregnant, planning to become pregnant, or nursing are also excluded from this study. The presence of any other active implanted device, such as a cardiac pacemaker or other implanted electromedical devices, and the presence of any other wireless sensor or transmitter located in the abdomen are also excluded. This study was registered in the clinicaltrial.gov: trial number: NCT06333366.

### Measurement tool and device

#### PressureDOT capsular intraluminal IAP measurement (IAPdot)

**Fig. 1:**
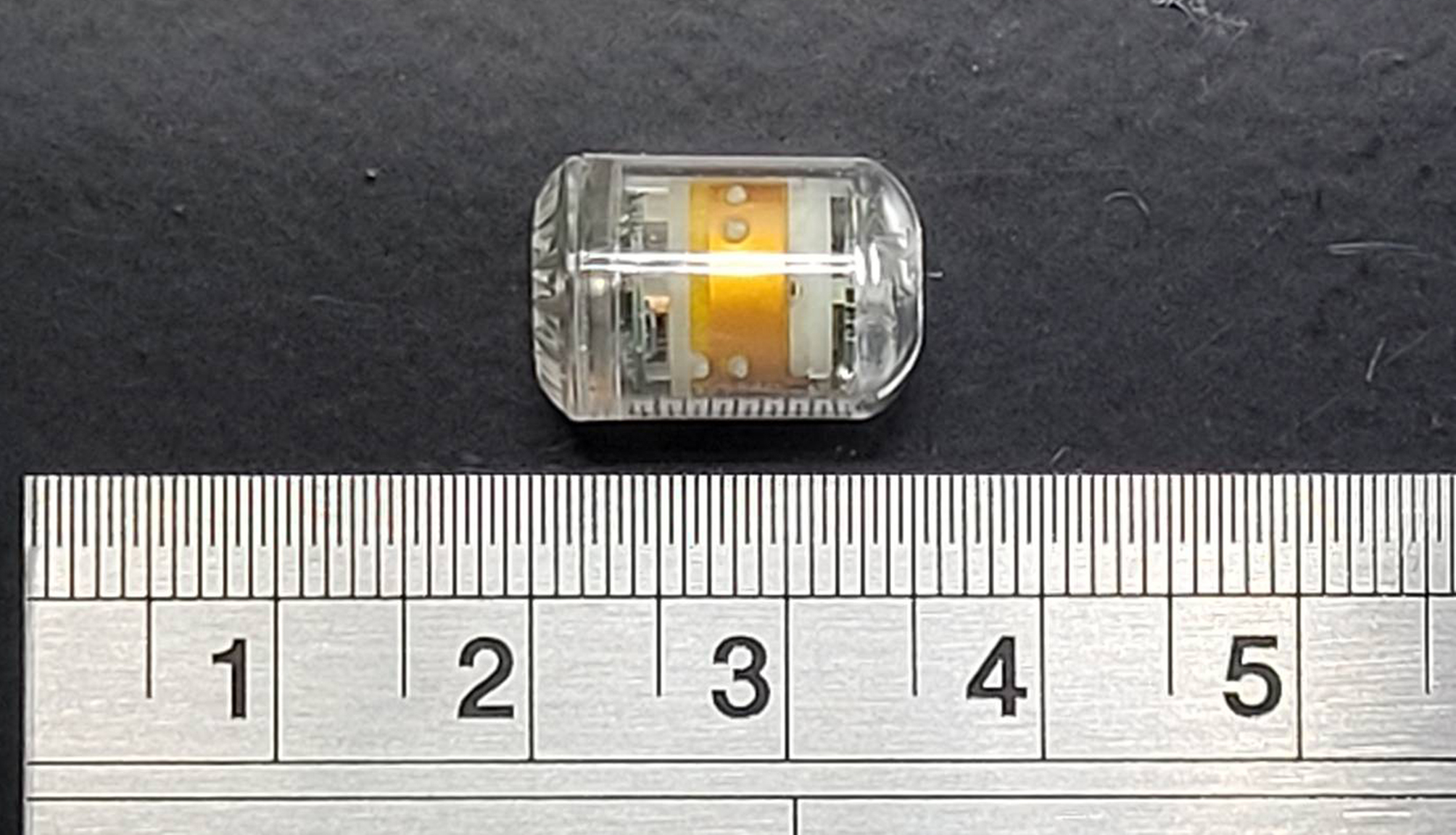
PressureDOT (PDT), measuring 12 mm in length and 6 mm in diameter, is equipped with both temperature and pressure sensors and capable of continuous working for 300 h

Intra-abdominal pressure was measured using the digital capsule: PressureDOT (PDT), a commercial medical device developed by Dotspace Inc. (Delaware, United States) (Fig. 1). This device is an ingestible capsule, measuring 12 mm in length and 6 mm in diameter, and is equipped with both temperature and pressure sensors. The device offers an accuracy of ± 0.5 °C for temperature and ± 0.5 mmHg for pressure. It has a battery life of 300 h and transmits data every 5 s via Bluetooth 5.0 to an external receiver connected to a laptop.

#### Intravesical pressure IAP measurement (IAPivp)

Following bladder emptying under anesthesia, a latex urinary dwelling catheter was inserted transurethrally and connected to a peristaltic pump for saline infusion. Pressure data were captured using a Nexam Pro (LABORIE, Toronto, Canada).

### Preoperative assessment and protocol

The participants received the first phantom capsule before the test.The phantom capsule is an equal-weighted device of the same outer shell but without the electric circuits. After the phantom capsule has been passed out, the participant will receive the standard PDT to record measured information continuously. The date of the laparoscopic surgery should be within follow-up day 1 or 2. We received the signals since the patient ingested the PDT till it passed out. Standard clinical intravesical pressure measurement will only be applied in the perioperative period of laparoscopic surgery. The participant will still be monitored by a standard PDT capsule afterwards. After the PDT capsule passed out, the participant will receive a complete checkup again for comparison.

### Perioperative protocol

**Fig. 2:**
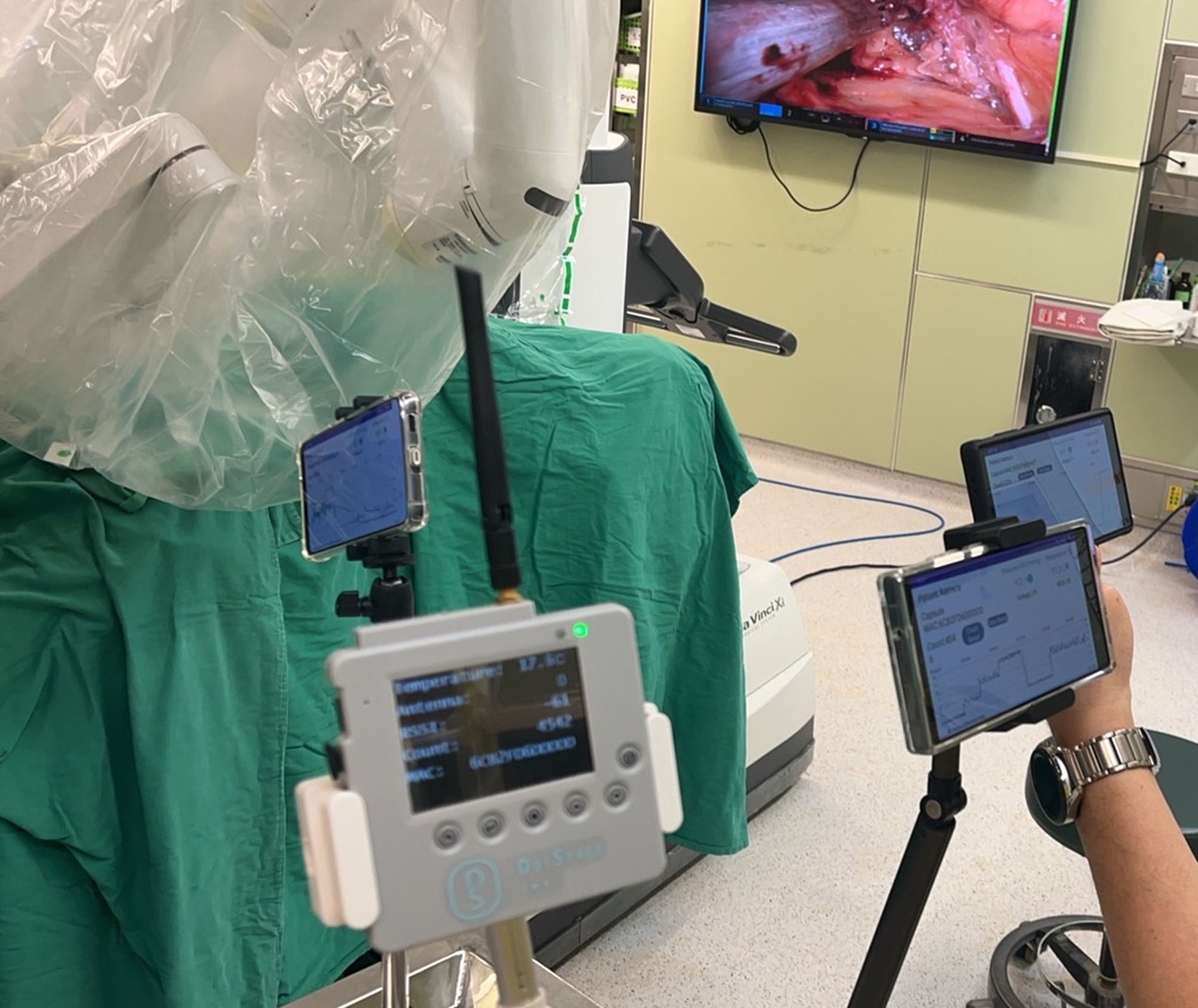
Multiple Bluetooth receivers were used to collect pressure data at different positions to complete all the transmission tests and the continuous IAP monitoring tests

During the perioperative period, the patients were under general anesthesia and prepreparement was done as routine work. The urinary dwelling catheter was applied for intravesical pressure data recording and monitoring. Following the administration of general anesthesia, the patient was prepared according to standard protocols. The surgeons and participants were not blinded to the procedure. Intraperitoneal inflation was initiated to gradually elevate IAP, starting from 0 mmHg. The inflating pressure was incrementally increased by 3 mmHg, maintaining each pressure level for a duration of 3 min. This process continued until the IAP reached a maximum of 15 mmHg, where it was held for an additional 3 min, the surgical procedure commenced. During the operation, data acquisition was paused. Upon completion of the surgery, IAP measurements were recorded again during the deflation phase. IAP data monitored from PDT (IAP_dot_) and IAP data monitored from urinary catheter (IAP_ivp_) were recorded and paired (Fig. 2). Additionally, we documented the duration of PDT retention within the patient, along with any instances of retention. Patient feedback was collected prior to discharge, and all adverse events were thoroughly documented. The length of hospital stay, as well as morbidity and mortality rates, were also systematically recorded.

### Study endpoints

First end point is the feasibility of signal transmission. The total data received divided by total data transmitted will be defined as data completeness. In the current study, successful transmission was defined as the ability of the capsule to transmit at least one data per minute. Degradation of wireless signals due to body mass, gastrointestinal tract contents, and sensor position, may affect data integrity [32]. Successful continuous IAP monitoring is defined as complete procedure conducted as scheduled for the intended indication, with PDT actively functioning after pass out.

The secondary endpoint is to evaluate device-related adverse events (AEs) and the overall AE rate. The safety analysis set will include all patients enrolled in the study. Individual listings of AEs will cover gastrointestinal discomfort, constipation, diarrhea, dyspepsia, hemorrhage, and capsule retention. Additionally, the seriousness, duration, relationship to the study device, and severity of each AE will be recorded.

The third endpoint is to compare the correlation between IAP_dot_ and IAP_ivp_. We synchronized the IAP_dot_ and IAP_ivp_ measurements by time and analyzed the differences and correlations between the two routes.

### Statistic methods and sample size calculation

All the data was collected and analyzed. The data loss rate was calculated from the received signals decided from estimated signal delivery times The difference between both measuring devices (IAP_dot_ vs. IAP_ivp_) was also calculated and analyzed. To assess the agreement between these two methods for detecting IAP, we calculated the required sample size based on the intraclass correlation coefficient (ICC). The ICC was estimated using a two-way mixed-effects model, with the method as a fixed effect and individual participants as a random effect, focusing on absolute agreement between single measures rather than average measures. The initial pilot study, which enrolled the first three participants, was conducted to determine the effect size for the basis of the sample size estimation. There were 182, 170, and 143 data units of IAP produced by both methods for participant no.1, no.2, and no.3, respectively. The calculated ICC value was 0.8724 (95% confidence interval: 0.8417–0.8966), which was used as the effect size for further sample size estimation. The ICC value under the null hypothesis was set at 0.5, known as the threshold for adequate agreement [33, 34]. Given an alpha level of 5%, a power of 80%, and 80 measurements per participant in the subsequent formal test, a minimum sample size of 5 participants was required. Therefore, we recruited six participants into this trial to compare the difference between IAP_dot_ and IAP_ivp_. A Bland-Altman plot was used to compare the point-to-point differences. In order to compare the agreement and reliability between IAP_dot_ and IAP_ivp_, ICC was used for statistically analysis.

## Results

**Fig. 3:**
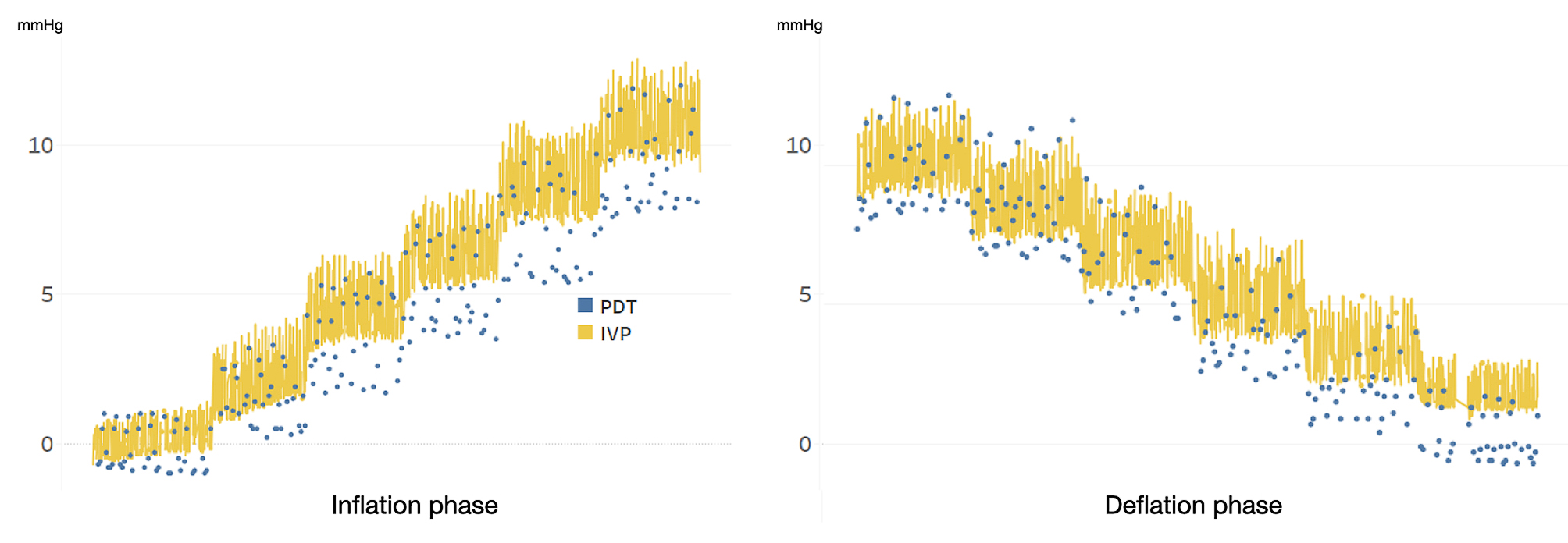
The change of intraabdominal pressure measurement during Inflation and Deflation phase of carbon dioxide. The intraabdominal measurement was performed by the device for urodynamic test (IVP, yellow line) and the PressureDOT (PDT, Blue dot)

By sample size calculation, we recruited six patients into this feasibility study. The inflation pressure ranged from 0 to 15mmHg. One of them underwent laparoscopic surgery and the other five underwent robotic surgery. The operative time ranges from 270 to 540 min with an average 264 +/- 84 min. There were no adverse events that took place, and all the patients recovered properly. No GI tract complication, inflammation or adverse event presented. All the capsules passed smoothly with well detection. The average excretion time was 88 +- 26.5 h. During the operation we inflated the peritoneal cavity smoothly and slowly and all the patients tolerated the operation. The average transmitted signal number PDT was 253.5 times. The characteristics of the patients were listed in Table 1. All the transmission tests and the continuous IAP monitoring test were passed. Since all of the patients were anesthetized, eliminating the influence of physical activity on intra-abdominal pressure fluctuations. Measurements were conducted over a 30-minute period (15-minutes inflation and 15-minutes deflation) with readings taken every 5 s. While this approach ensures reliable data collection, the short interval between measurements makes it difficult to attribute pressure changes specifically to peristalsis. The continuous monitoring data from PDT and IVP were shown as Fig. 3.

**Table 1:** The characteristics of patients who undergo this study

	Age	Gender	Op Type	Op time	Post-op intake time	PDT excretion time	Adverse event
Case 1	69	M	Robotic radical prostectomy	6.6 h	Within 24 h	72 h	nil
Case 2	80	M	Robotic radical prostectomy	4.5 h	Within 24 h	72 h	nil
Case 3	58	F	Robotic partial nephrectomy	6.1 h	Within 24 h	72 h	nil
Case 4	70	F	Laparoscopic partial nephrectomy	6.1 h	Within 24 h	72 h	nil
Case 5	48	F	Robotic partial nephrectomy	9.1 h	Within 24 h	96 h	nil
Case 6	64	F	Robotic partial nephrectomy	5.9 h	Within 24 h	144 h	nil
				6.4 +/- 1.4 h	Within 24 h	88 h +- 26.5	nil

**Fig. 4:**
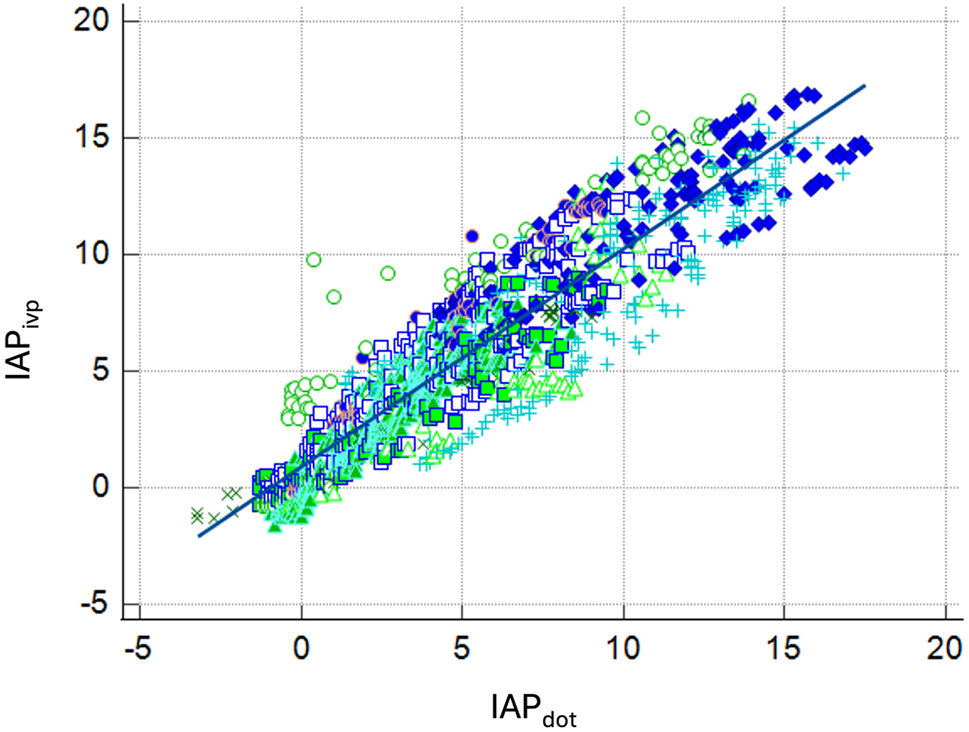
The summarized correlation dot plot to compare IAPdot and IAPivp

**Fig. 5:**
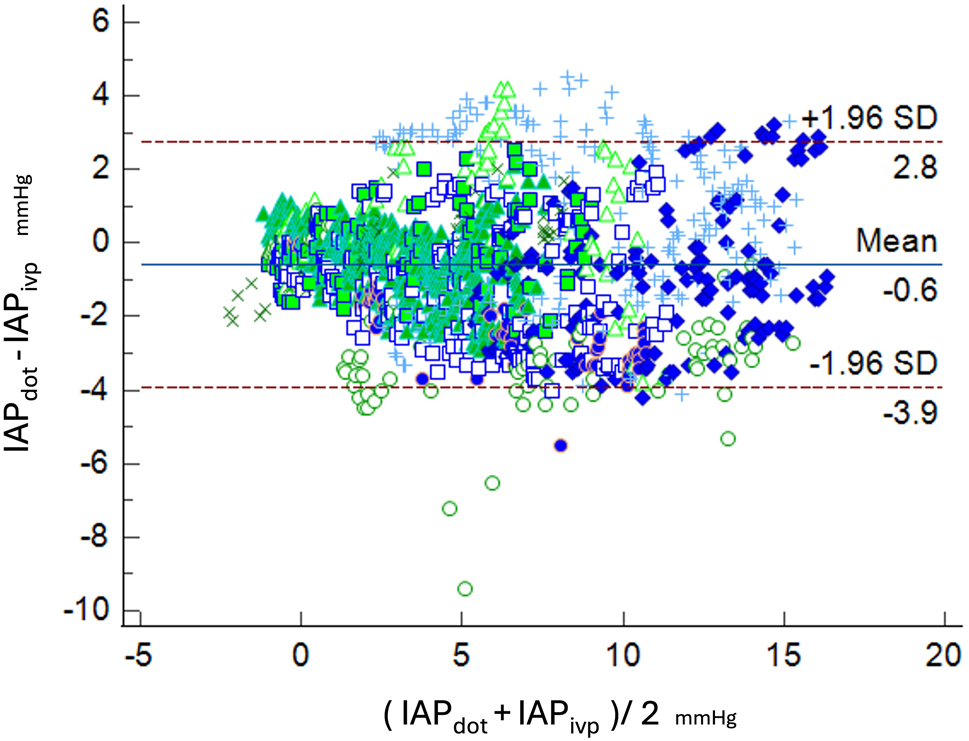
Bland and Altman’s analysis for the IAP_dot_ versus IAP_ivp_. The mean bias was − 0.57 mmHg, 95% Confidence interval: − 0,6617 ~ -0.4902

We synchronized IAP_dot_ and IAP_ivp_ by time and compared point to point. The correlation was similar in every individual participant and the ICC ranged from 0.8791 to 0.9678 (Table 2). And we summarized all participants paired- measurement together with 1521 paired IAP measurements. The correlation is similar, and the summarized ICC is 0.9119 (95% CI 0.8821 ~ 0.9320). The summarized correlation dot plot is depicted in Fig. 4. In the summarized data, the mean IAPdot is 0.6 mmHg lower than the IAPivp, with a standard deviation of 1.68 mmHg. And the Bland-Altman plot was demonstrated as Fig. 5 Table (2).

**Table 2:** The transmitted signal and the intraclass correlation between IAP_dot_ vs. IAP_ivp_

	Transmitted data pair	Continuously IAP monitoring achievement	Single measure ICC	95% Confidence interval
Case 1	182	yes	0.9255	0.8451 ~ 0.9578
Case 2	170	yes	0.9123	0.8831 ~ 0.9344
Case 3	143	yes	0.9124	0.8740 ~ 0.9383
Case 4	244	yes	0.9678	0.9215 ~ 0.9830
Case 5	462	yes	0.9360	0.8658 ~ 0.9632
Case 6	320	yes	0.8791	0.5300 ~ 0.9482
summarized	1521	yes	0.9119	0.8821 ~ 0.9320

The Bland-Altman plot was drafted as Fig. 5.

## Discussion

In this study, we did the first-in-human study to prove the IAP monitor by ingestible electric capsule, which can offer continuous and immediate data which can assist clinical doctors to monitor the IAP of patients and possibly associated IAH and ACS. We found PDT can perform this work well and transmit the IAP data with acceptable transmission rate. There were also no adverse events in our participants and all of the PDTs passed out smoothly. As an essential physiologic parameter, the IAP measurement plays an important role in critical care, especially in trauma, burn, post-transplantation and cardiothoracic surgery patients [35, 36]. In this study, we found the PDT offered continuous data in patients who underwent changing IAP by inflation during laparoscopic surgery. IAP_dot_ offered an immediate response of pressure shift rather than IAP_ivp_. Another comparison in this study is to evaluate the difference between IAP_dot_ and IAP_ivp_. Several capsular devices are designed to monitor the contractile pressure for the alimentary tract. The provided additional usage to monitor IAP in critical care practice by the transparency of pressure through the digestive tract wall, the PDT can also detect IAP continuously. We compared the difference between both methods and noted the difference in the range between 2mmHg with a p value < 0.05. The summarized paired data showed the R2 is above 0.95 with a significant correlation between IAP_dot_ and IAP_ivp_ and the interclass correlation is 0.9668, also with significant difference.(*p* < 0.001). The high correlation between IAP_dot_ and IAP_ivp_ makes us believe the PDT is a good route to measure IAP directly. In previous experience, we did animal studies to prove the feasibility and accuracy of digital capsules to measure the IAP in an animal setting [30]. In this study, we performed the clinical trial to evaluate the reliability between IAP_dot_ and IAP_ivp_. As a result, this is the first in human study to prove the concept of IAP monitoring but a digital capsule and did an adequate comparison with conventional routes from urinary bladder pressure monitoring. We can extend the usage of digital capsules in different practices, especially for critical care.

Currently, the most common method of measuring IAP is a measurement of hydrostatic pressure within the urinary bladder. However, there were several drawbacks of this method [37]. One of the most important, the precision of the method is insufficient [38], the 95% limits of agreement of value measured intravesical and laparoscopically are wide for a useful measurement to be assumed and outside of guidelines of acceptable accuracy [39]. Reliability is also dependent on the staff conducting the procedure and this can lead to significant discrepancies between measurements [21, 22]. Furthermore, the method is daily labor-intensive and is usually only applied intermittently [23]. Several devices other than IVP exist for measuring IAP, and previous studies have demonstrated a correlation between different measurement routes [40]. Pressure within the GI tract results from the intraluminal pressure caused by changes in abdominal pressure and the contractile pressure due to peristalsis [26, 41]. Since the frequency of peristalsis is limited, alternative routes like the GI tract can be considered for measuring IAP. The stomach has about 3 contractions per minute, while the intestines range from 6 to 9 contractions per minute. In contrast, the colon can exhibit as slow as one contraction every 30 min [42]. An in vitro study confirmed that measurements from the intragastric pressure and IAP_ivp_ were similar [43, 44]. Another study further support the similarity between intragastric pressure and IAP_ivp_ in measuring IAP [45]. However, no prior clinical studies have provided continuous monitoring of IAP by wireless digital capsule in an adjustable IAP environment. In current study, we adjusted intra-abdominal pressure by inflating CO2 into the abdominal cavity of patients undergoing minimally invasive surgery. We found that PDT measurements accurately and precisely reflected immediate changes in IAP corresponding to the inflated pressure. In contrast, IVP measurements, which are derived from hydrostatic pressure in the bladder, did not clearly show any delay in pressure presentation. By PDT, the precision and accuracy between IAP_dot_ and IAP_ivp_ were properly correlated. Once the PDT was delivered or swallowed into the patient’s GI tract, continuous monitoring persisted for more than 144 h with no necessary additional labor-effort (as supplementary 1, unpublished information). There was no conducting error and bias, no associated calibration needed that made the reliability increased. Without reflux fluid back to the urinary bladder, there was no increasing opportunity for urinary tract contamination or infection, and the patient can remove the Foley dwell device as soon as possible and keep monitoring their IAPs. Another issue raised is the difference of pressure measured values were similar in all tracts of the GI tract or not. As in the previous publication [46, 47], the baseline pressures were similar but the contractile of GI tract will influence the pressure change. As discussed above, once the peristaltic frequency is limited, we can account for any bias introduced by unexpected, elevated pressure levels from peristalsis and still achieve accurate IAP monitoring. This correction allows us to filter out the influence of peristalsis and obtain reliable data.

We mentioned that the ingestible device offers real-time and continuous data, which far exceeds the capabilities of current clinical practice. Similar to other monitoring systems, we anticipate that continuous and real-time monitoring of physiological parameters will enhance our ability to accurately assess patients. In current practice, conventional urinary catheters are used, particularly in critical care settings. Real-time pressure transmission and parameter presentation can significantly improve the quality of care [48]. Just as with arterial and venous pressure monitoring, real-time IAP monitoring not only provides immediate IAP data but also accesses the patient’s response to treatment, thereby facilitating prompt therapeutic decisions. The pressure varies according to the patient’s condition. Even if the patient’s IAP is not initially above the intra-abdominal hypertension threshold, it may increase within minutes to hours. In previous measurement practices, IAP was typically recorded at intervals of every four to eight hours. It is possible that during these scheduled measurements, the patient’s pressure was either within normal limits or at a borderline level. However, in the intervening hours between measurements, the pressure could persistently exceed the threshold, potentially leading to undetected organ ischemia and subsequently increasing the risk of complications. Today, advanced techniques are available to provide continuous IAP (CIAP) measurement and estimation [10]. Although CIAP can be directly measured via the peritoneum, this method is not recommended for critically ill patients due to its invasiveness and associated risks, including infection and complications. Balogh et al. proposed an alternative method for continuous IAP measurement using a three-way Foley catheter, demonstrating strong correlation with intermittent IAP measurements [49]. Nevertheless, this technique does not eliminate the risks of regurgitation from continuous irrigation or the occurrence of catheter-associated urinary tract infections, which remain significant concerns. PDT offers an ultimate route to detect CIAP and provides continuous APP trends, which may serve as a more effective resuscitation target and facilitate early detection of impending ACS. In addition, IAP serves as a prognostic marker in surgical patients during exploratory laparotomy, with higher numbers associated with mortality, a higher SOFA score and prolonged ileus [50]. In cardiac surgery patients, High incidence of elevated IAP in cardiac surgery patients was noted during the first two hours in the ICU. The use of CIAP enables earlier treatment adjustments, particularly in postoperative patients with risk factors. Furthermore, CIAP monitoring facilitates automated data processing, enabling the calculation of metrics for further morbidity and mortality prediction and management [10].

### Limitation

Our study presented a feasibility study to show the efficiency and reliability to use digital capsules to detect intraabdominal pressure continuously in patients with alteration of intraabdominal pressure during minimally invasive surgery. While the findings demonstrate the potential efficiency and reliability of this method, several limitations should be acknowledged. First, the small sample size may have constrained the study’s power to fully assess the tool’s feasibility. The initial three cases were treated as pilot cases to inform sample size calculations and ensure the recruitment of an adequate number of cases based on rigorous statistical analysis. Second, the study’s setting differed from typical clinical scenarios where CIAP monitoring is required. Specifically, we adjusted IAP using CO2 insufflation during surgery, a condition that does not precisely replicate the IAH scenarios commonly encountered in clinical practice. This study was limited to demonstrating the agreement between IAP measurements from PDT and IAPivp within a near-normal pressure range, rather than clinically relevant ranges where patient outcomes may be impacted. In critically ill or unconscious patients, the capsules were designed to be delivered via orogastric tube. However, in this study, all capsules were swallowed prior to the operation. Future clinical trials, particularly in ICU patients, are necessary to further validate this approach. Further research in critical care settings is necessary to validate the method across clinically significant IAP ranges. The data on IAP in awake patients and the pressure change along the whole GI tract are indeed of interest (supplementary 1). However, the primary objective was to replicate clinical practice conditions where IAP monitoring is typically performed with patients in a sedated, supine position at the end of expiration. Therefore, we did not have direct results in the current study. Another limitation of this study is that the operator reporting the measurements was not blinded, which could introduce potential bias in the data collection process. Future studies should consider implementing blinding to minimize such bias and strengthen the validity of the findings.

## Conclusion

In this feasibility study, we found the digital capsule can measure IAP_dot_ continuously with good correlation with conventional IAP_ivp_. WIth the support of real-time and continuous monitoring, the digital capsule can offer additional benefits than routine monitoring IAP and provide an efficient method to check IAP as possible.

## Electronic supplementary material

Below is the link to the electronic supplementary material.

Supplementary Material 1

Supplementary Material 2

## Data Availability

Data supporting the findings of this study are available for research purposes upon request to the corresponding author due to institutional review board request.
