# Supplementary figures and images for: Feasibility and accuracy of continuous intraabdominal pressure monitoring with a capsular device in human pilot trial
Source: World J Emerg Surg. 2025 Jan 27;20:7. doi: 10.1186/s13017-024-00569-0 (PMC11771060; doi:10.1186/s13017-024-00569-0)

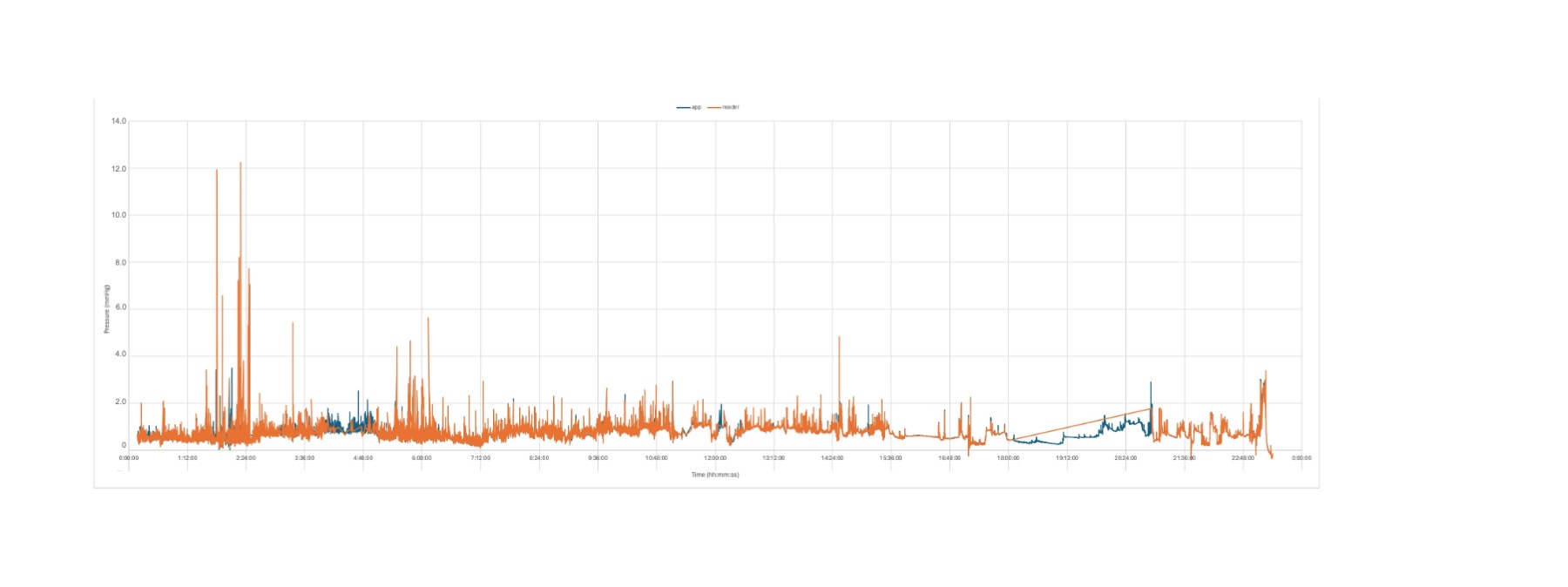

Supplement: Supplementary file 1 — Supplementary Material 1 [file 13017_2024_569_MOESM1_ESM.jpg]
