# Supplementary material for: Feasibility and accuracy of continuous intraabdominal pressure monitoring with a capsular device in human pilot trial
Source: World J Emerg Surg. 2025 Jan 27;20:7. doi: 10.1186/s13017-024-00569-0 (PMC11771060; doi:10.1186/s13017-024-00569-0)
Supplement: Supplementary file 2 — Supplementary Material 2 [file 13017_2024_569_MOESM2_ESM.docx]

**Supplementary illustration for 24 hour IAP monitoring.**

This supplement figure represents the continuous intra-abdominal pressure (IAP) data recorded over a 24-hour period. This is an unpublished data which is for initial concept proof.

The orange line illustrates the IAP measurements received via the receiver, while the blue line reflects the data collected through the app. The variance in IAP readings is primarily associated with patient movement, indicating periods of physical activity throughout the monitoring duration.

Although fluctuations are evident, it is important to note that the variance remained within 2 mmHg throughout the monitoring period, demonstrating a relatively stable IAP profile even during periods of movement. During the first few hours, there is noticeable fluctuation in IAP, with several peaks reaching above 10 mmHg. These variations are likely due to movement and physical activity, which commonly influence IAP values. As the recording progresses, a distinct pattern emerges, where periods of reduced variance correspond with likely times of rest or sleep. During sleep, the IAP changes are minimal, further emphasizing the stability of intra-abdominal pressure when the patient is immobile. The contrast between the high-frequency changes during wakefulness and the minimal fluctuation during sleep highlights the impact of physical activity on IAP measurements.

Notably, around 12 to 15 hours into the monitoring period, both the receiver and app data display a marked decrease in IAP fluctuation, indicating a prolonged period of rest. Toward the end of the monitoring session, another spike in IAP is observed, which may correlate with the resumption of physical activity or other factors influencing abdominal pressure.

Overall, this figure demonstrates the dynamic nature of IAP in response to patient activity and underscores the importance of considering physical movement when interpreting continuous IAP measurements over an extended period. The observation that variance remained within 2 mmHg, despite fluctuations, provides further evidence of the reliability of the monitoring system used.
